# Evaluation of ocular surface microbiota in children with blepharoconjunctivitis

**DOI:** 10.1007/s00417-025-06836-3

**Published:** 2025-04-23

**Authors:** Burçin Çakır, Büşra Güner Sönmezoğlu, Elif Özözen Şahin, Mehmet Köroğlu, Nilgün Özkan Aksoy

**Affiliations:** 1 Department of Ophthalmology, Sakarya University Faculty of Medicine, Sakarya, Turkey; 2 Department of Ophthalmology, Serdivan State Hospital, Sakarya, Turkey; 3 Department of Clinic Microbiology, Sakarya Training and Research Hospital, Sakarya, Turkey; 4 Department of Clinic Microbiology, Sakarya University Faculty of Medicine, Sakarya, Turkey

**Keywords:** Blepharoconjunctivitis, Microbiota, Ocular surface, Pediatric age group

## Abstract

**Purpose:**

To investigate the conjunctival and eyelid margin bacterial microbiota in children with blepharoconjunctivitis by using 16S rDNA amplicon sequencing.

**Methods:**

In this prospective cross-sectional study, 20 children aged between 3–15 years with blepharoconjunctivitis or blepharokeratokonjunctivitis formed Blepharitis Group and 21 children aged between 3–15 years without any ocular and sysemic diseases except mild refractive errors formed Control Group. Swap samples from all children were taken. The alpha diversity of the ocular surface microbiota within each group were evaluated by using Shannon's, Simpson, and Chao index. Beta diversity was evaluated by Bray Curtis index.

**Results:**

Microbiological diversity was higher in the patient group than in the control group. According to Shannon's, Simpson, and Chao index, there were statistically difference between groups (p: 0.000013, p:000003 p: 0.00235, respectively). According to the Bray Curtis index, the healthy eye microbiome in the control group is observed to be highly similar, consistent with other analyses, and the overlapping cluster with the blepharitis eye microbiome is quite low (pco1: 40.93%). *Sphingoblump, Micrococus*, *Lacnospiracebacterium, Stenothermophilus, Aurelmonass, Micrococus, Blatiabeum, Delfiacdiovorans* and *Vellonella* densities were found to be higher in the patient group.

**Conclusion:**

Both alpha and beta diversity analyses were significantly higher in pediatric age group patients with blepharitis. In addition, *Lacnospiracebacterium, Stenothermophilus, Aurelmonass, Micrococus, Blatiabeum, Delfiacdiovorans* and *Vellonella* densities were found to be higher, which may lead to future studies focused on diagnosis and treatment.

## Key messages

***What is known***

•In studies based on culture results from eyelids and conjunctiva, Staphilococcus aureus, Staphilococcus epidermidis and Propionium acnes have been found to be common organisms in children with blepharitis.

• In a study based on 16S rDNA amplicon sequencing, it was shown that *Proteobacteria* is dominated the ocular flora of healthy children.

• According to 16S rDNA amplicon sequencing analysis, *Lactobacillus*, *Bifidobacterium*, *Akkermansia*, *Ralstonia*, and *Bacteroides* may play important roles in the pathogenesis of blepharitis in adults.

***What is new***

• Both alpha and beta diversity analyses were significantly different in pediatric age group with blepharoconjunctivitis compared to the healthy control group.

• Some bacteria detected on the ocular surface in adult blepharitis and some diseases affecting the ocular surface such as type 1 DM and Sjögren's Syndrome were also seen intensively in the pediatric patient group.

• *Lacnospiracebacterium*, *Stenothermophilus*, *Aurelmonass*, *Micrococus*, *Blatiabeum*, *Delfiacdiovorans* and *Vellonella* densities were found to be higher in children with blepharoconjunctivitis.

## Introduction

Blepharoconjunctivitis (BC) of childhood is a chronic inflammatory disease of the palpebral margin and secondary conjunctival and corneal involvement is not uncommon. The disorder has a wide range of clinical manifestations, including chronically inflamed eyelids, meibomian gland dysfunction, lid margin telangiectasias, collarettes at the bases of the eyelashes, recurrent chalazia, conjunctivitis, keratopathy, and even amblyopia and vision loss [1].

The main pathophysiological cause of BC is inflammation. Bacterial colonization of the conjunctiva and eyelids with Staphylococcus aureus, Propionibacterium acnes, or Corynebacteria may stimulate the release of inflammatory cytokines from the ocular surface and destabilize the tear film via release of free fatty acids by bacterial phospholipases [1]. Studies have been performed to find out bacteria species, in childhood. All of these studies evaluated the culture results taken from lid and conjunctival tissues. Staphilococcus aureus, Staphilococcus epidermidis, and Propionium acnes were found to be common organisms [2–5]. However, culture conditions itself, might alter the true composition of the microbiota. In recent years, as a culture-independent approach with high sensitivity and specificity, high-throughput sequencing technology has been considered more appropriate to analyze the human microbiota than traditional culture methods, and the relative abundances of tens or even hundreds of times more bacteria can be identified [6]. In a recent study; Lactobacillus, Bifidobacterium, Akkermansia, Ralstonia, and Bacteroides were found to play important roles in the pathogenesis of blepharitis, in adult patients [7].

In this prospective study, the conjunctival and eyelid margin bacterial microbiota is investigated in children with blepharoconjunctivitis by using 16S rDNA amplicon sequencing. To our knowledge, this study is the first which evaluated ocular surface microbiota in pediatric age group with BC and can help to clarify the pathogenic mechanism of blepharitis in children.

## Methods

This prospective cross-sectional study was conducted at Sakarya University Educational and Research Hospital. Prior approval was taken from the Institutional Review Board (Ethical Committee of Sakarya University, Faculty of Medicine, IRB) (IRB number: E- 16214662–050.01.04–119926 - 33), and informed consent was taken from the parents of each subject. This study was funded by Sakarya University Medicine Faculty Scientific Research Project Support Unit, and in compliance with the principles of the Declaration of Helsinki.

The records of children diagnosed with chronic blepharoconjunctivitis and/or blepharokeratoconjunctivitis from January 2019 to January 2022 were analyzed. According to this investigation, the Blepharitis Group was determined as 20 children aged between 3–15 years. Topical antibiotics and steroids had been discontinued six weeks prior to conjunctival swab collection. Therefore, the patients were in the post-critical phase of the disease. Inclusion criteria were; at least 3 month clinical follow-up time, the cessation of drugs including antibiotics, corticosteroids, cytokines, immunosupressive agents, metformin, proton pump inhibitors, and probiotics if used, at least six weeks before conjunctival swab sampling, and no additional ocular and systemic disease presence. In order to prevent changes in ocular microbiota, diseases that may affect ocular microbiota such as cutaneous form of rosacea, type 1 diabetes mellitus (DM), autoimmune diseases have been questioned in detail.

The control group consisted of 21 children aged 3–15 years without any ocular and systemic disease except mild refractive errors (hyperopia, myopia or astigmatism up to 1 diopter) in order to obtain an age, gender and numerically matched group. If used at least six weeks before conjunctival swab sampling, drugs including antibiotics, corticosteroids, cytokines, immunosupressive agents, metformin, proton pump inhibitors, and probiotics were stopped.

Swab samples from children in the Blepharitis Group were taken from one eye with confirmed blepharitis. In the Control Group, sampling were performed from one random eye. The children were asked to look up. Because of the irritating pattern of topical agents, topical anesthesia was not used. While talking and distracting the attention of child by the help of parents, a disposable aseptic cotton swab was used to wipe the lower conjunctival sac from the nasal to temporal side and backwards while rotating the swab. Utmost attention was paid to avoid sample contamination from the eyelashes or outer eyelid skin. Each swab was immediately placed into a sterile tube and stored in an ultralow-temperature freezer at − 80 °C before DNA extraction.

The primer pair to be used to create amplicon libraries targets a region of approximately 1500 bp covering the V1-V9 region of the 16S rRNA gene. Oxford Nanopore Technologies Nanopore barcode DNA sequences of the created library were added to the 5'ends of the target-specific primer pairs. Target-specific primer-connector sequences specific to 16S rRNA are TTTCTGTTGGTGCTGATATTGC- AGRGTTTTGATYHTGGCTCAG − 3'for the forward primer and 5'-ACTTGCCTGTCGCTCTATCTTC- TACCTTGTTAYGACTT − 3'for the reverse primer. The first PCR was performed using Proof Reading DNA Polymerase 2 × Reaction Mix and 200 nm of each primer. The following thermal cycling program was followed in the PCR device: 3 min at 95 °C; 25 cycles of 30 s at 95 °C, 30 s at 55 °C, and 90 s at 72 °C; 5 min at 72 °C. The PCR product was run on agarose gel to verify its size (~ 1450 bp) and was purified using the PCR Product Purification Kit.

Ligation sequencing kit (SQK-LSK109; Oxford Nanopore Technologies) and Native barcoding kit (EXP-NBD104 - 114; Oxford Nanopore Technologies) protocols were used to prepare the amplicon library. First, the ends of the samples (100–200 fmol) in a final volume of 60 uL were prepared, dA tail was added and repaired. Afterwards, 0.5X MagBeads (MobiomX) kit was used for purification. The native barcodes were kept at room temperature for 10 min to attach to the prepared tips. The samples were then purified and measured spectrofluorimetrically.

Equal amounts of DNA were taken from each of the barcoded samples and combined in a single tube to reach a final volume of 35 μL. Adapters were ligated to the ends of barcoded samples, purified, and measured. After preparation, 50 fmol of the library was loaded into a Spot-On flow cell (FLO-MIN106D). The sequencing run was initiated using MinKNOW™ software on the Mk1 C™ device (Oxford Nanopore Technologies). Sequencing was stopped when sufficient data was obtained or the maximum run time of 72 h was completed.

After the sequencing process, the results obtained in fast5 format were converted to fastq format using guppy software (base-calling and de-multiplexing). Since the 16S rRNA region averages 1500 bp, reads 1250–1750 bp long were filtered using Trimmomatic and the remaining reads were excluded from analysis. Cleaned reads were analyzed with a customized workflow using the python programming language. With this workflow, each sequence was matched with the BLAST algorithm during the filtering process. An operational taxonomic units (OTUs) were created by taking the taxonomic data of sequences with more than 60% reference coverage and 80% pairwise similarity in the matching results.

In order to perform phylogenetic analyzes with the created OTU (.biom) file, alpha diversity analysis, PCA, PCoA, beta diversity analyses, biomarker and phenotype analyzes were performed using different indexes with the tools provided by the QIIME2 platform. The Mothur platform was used to organize taxonomic classifications and prepare dynamic chrono charts. The graphs and tables included in the analyzes were made with libraries of the Python programming language.

Linear discriminant analysis effect size (LEfSe) was used to identify bacterial biomarkers of each group. The Mann–Whitney U-test and Chi-square test was used to compare the differences in age and sex between the patients with blepharitis and the controls. The Mann–Whitney U-test was performed for analyses of the α-diversity indices and the relative abundances of dominant phyla and genera among different groups. For principle coordinate analysis (PCoA), the permutational multivariate analysis of variance (PERMANOVA) statistical method was used to compare the differences. Statistical analyses were carried out with SPSS 26.0 (Chicago, IL, USA) software, and *p* < 0.05 was considered to be statistically significant.

## Results

There were no statistical differences between blepharitis and control groups in terms of age, gender, best corrected visual acuity, spherical equivalent and astigmatism. Table 1 reveals characteristics of both groups. Ocular surface findings of children with blepharitis are summarized in Table 2.

**Table 1:** Characteristics of children in Blepharitis and control groups

	Blepharitis group	Control group	*p*
Mean age (years)	8,80 ± 3.10	8,14 ± 2,86	0,544
F/M	15/5	13/8	0,368
Mean BCVA	0,91 ± 0,10	0,96 ± 0,06	0,156
Mean SE (D)	0,30 ± 2,53	0,14 ± 0,72	0,724
Mean astigmatism (D)	0,03 ± 1,22	0,01 ± 0,44	0,355

*F* Female; *M* Male; *BCVA* Best corrected visual aquity; *SE* Spheric equivalan

**Table 2:** Ocular surface findings in children with blepharitis (n:20)

Ocular surface findings	%
Peripheral corneal opacity	50
Peripheral corneal vascularization	25
Previous multiple chalasion history	30
Corneal punctate epitheliopathy	95

**Fig. 1:**
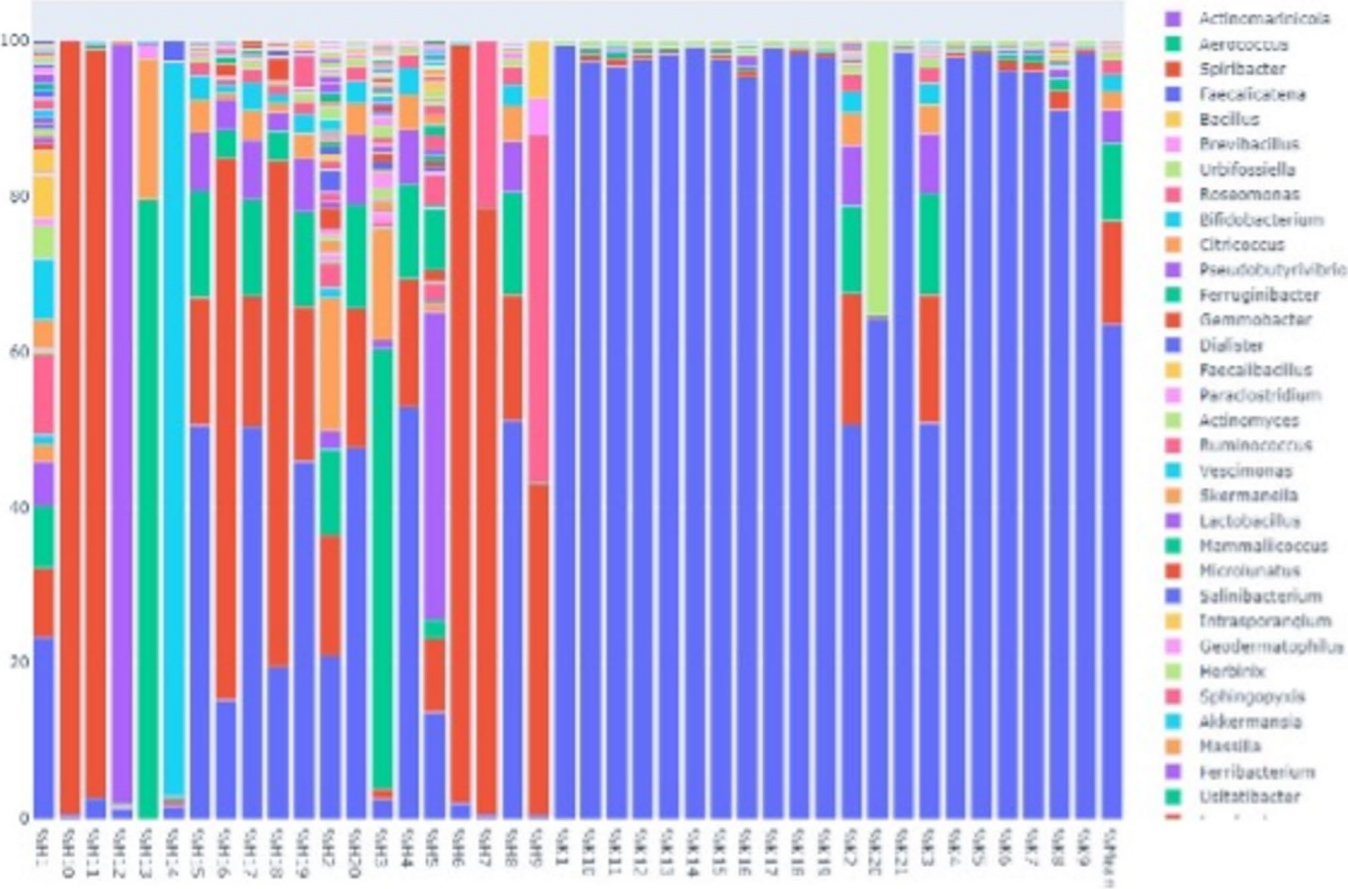
The relative abundance graph, in patient and control groups, respectively

**Fig. 2:**
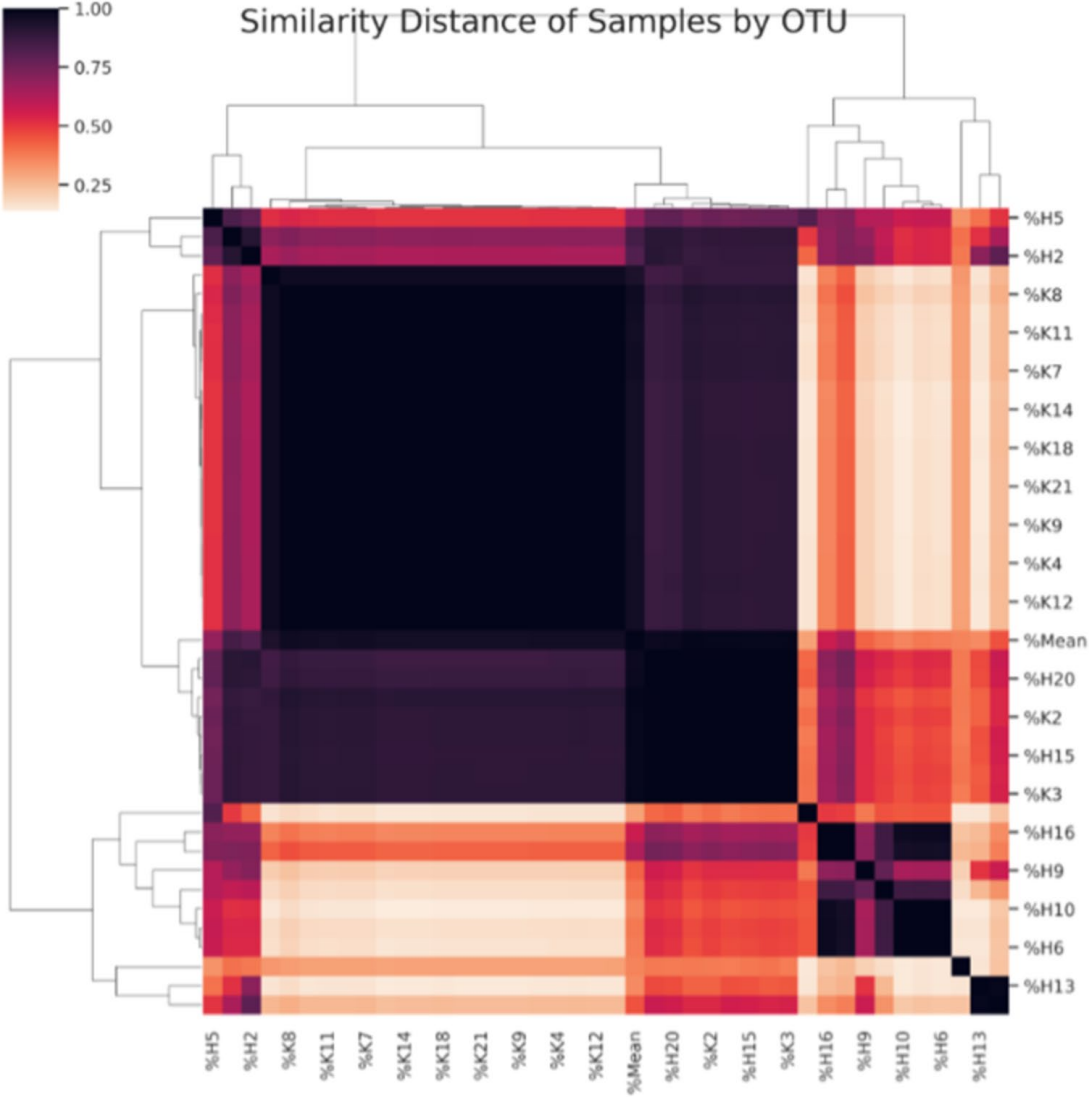
The dendogram created by diversity and quantity of samples

The relative abundance graph shows that microbiological diversity was higher in the patient group than in the control group (Fig. 1). The dendogram shown in Fig. 2 was created by analyzing the proximity of the samples to each other according to their diversity and quantity. One of the samples is indicated as “mean”. This sample was created by averaging all the OTUs, expressed as a percentage, and is included to represent the average diversity. The control group is very similar in diversity.

**Fig. 3:**
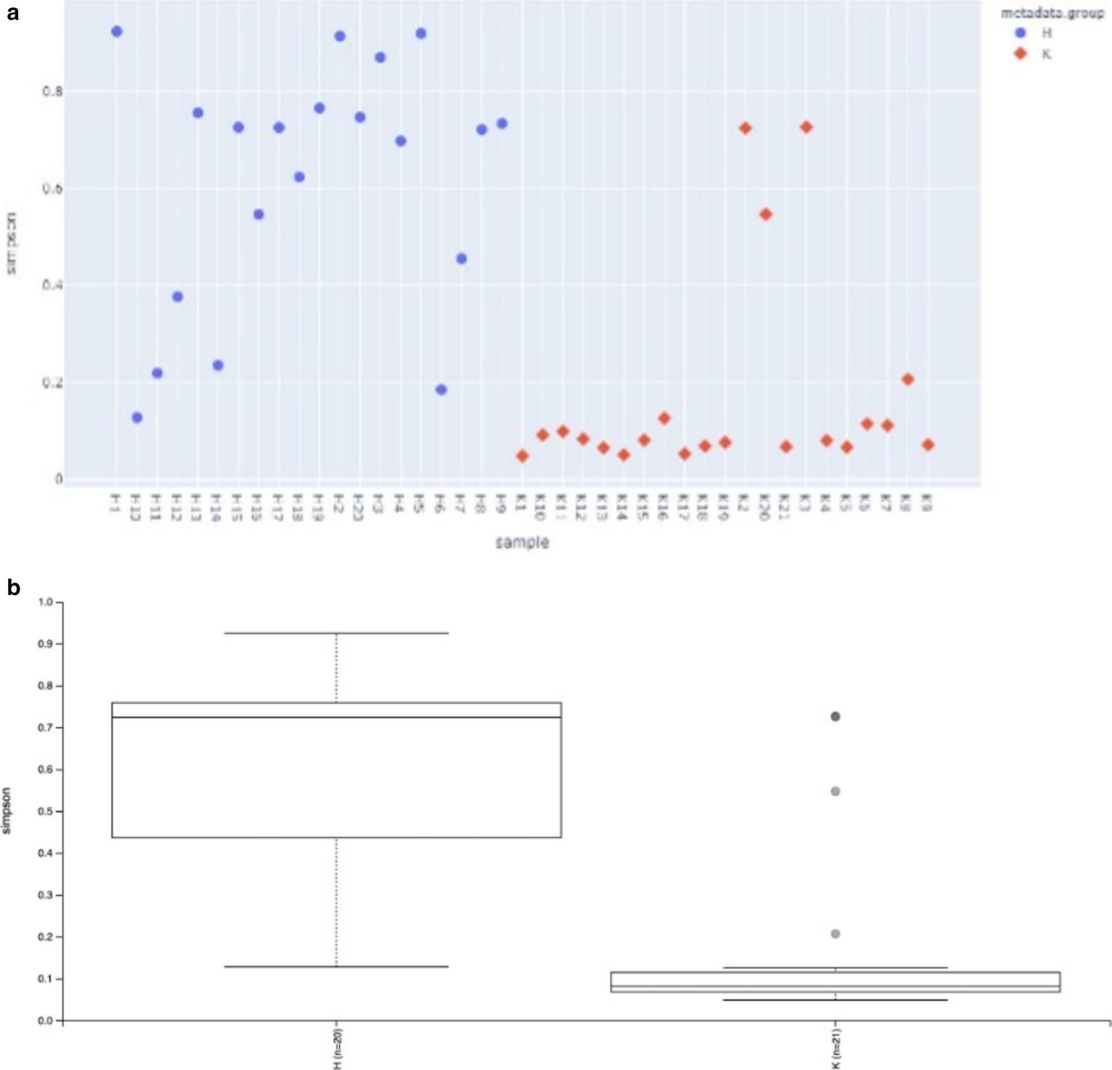
**A** Alpha diversity analysis graph. **B** Alpha diversity analysis Box-plot graph

The alpha diversity (Shannon index, Simpson, and Chao index) of the ocular surface microbiota within each group were calculated and evaluated. According to Shannon's, Simpson, and Chao index, there were statistically difference between groups (p: 0.000013, p:000003 p: 0.00235, respectively) (Fig. 3A-B).

**Fig. 4:**
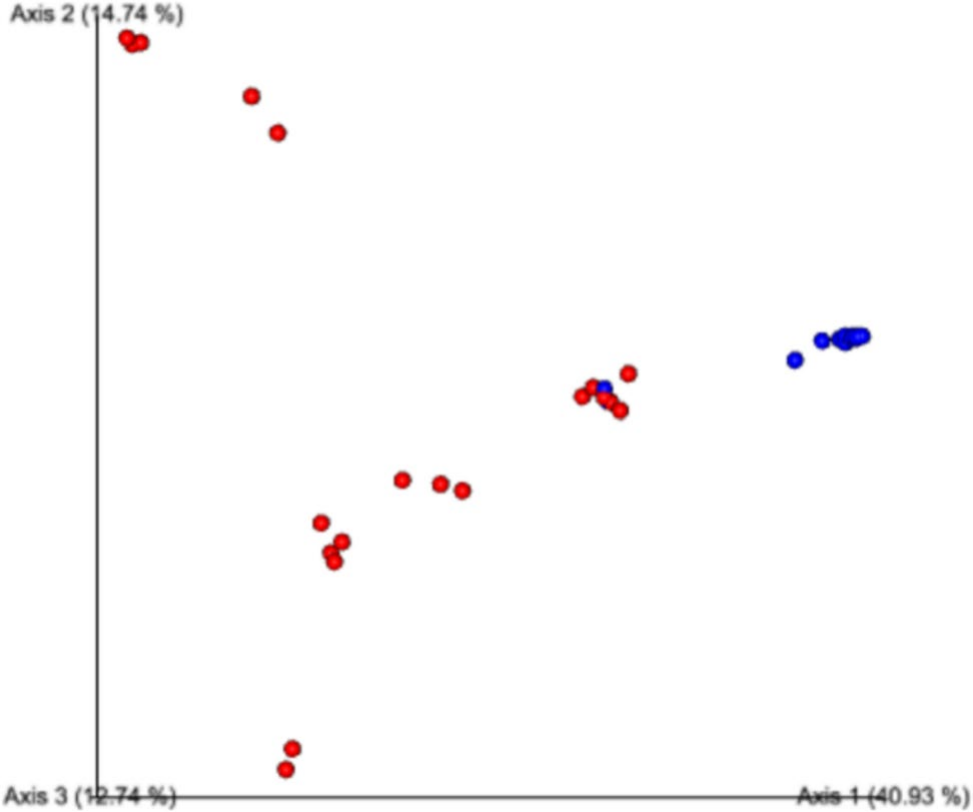
Beta diversity analysis according to Bray- Curtis analysis (Blue dots: ocular surface microbiota of control group, red dots: ocular surface microbiota of patient group)

According to the Bray Curtis index, where beta diversity is evaluated, the healthy eye microbiome in the control group is observed to be highly similar, consistent with other analyses, and the overlapping cluster with the blepharitis eye microbiome is quite low (pco1: 40.93%) (Fig. 4).

**Fig. 5:**
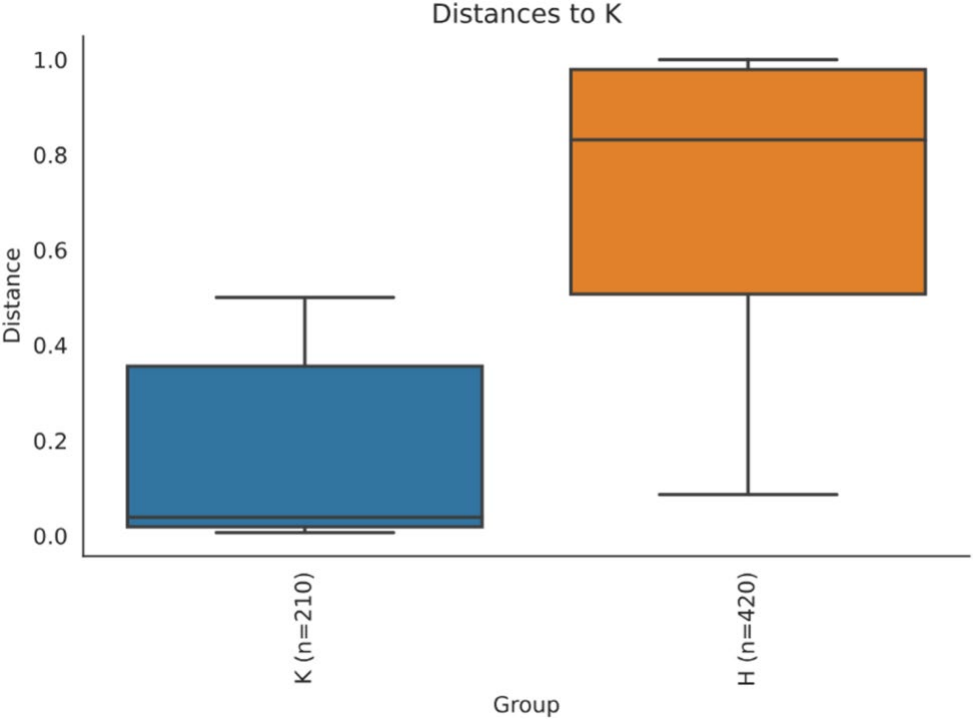
Boxplot graph of beta diversity according to Bray–Curtis index

The boxplot graphics of beta diversity analysis according to the Bray–Curtis index is also revealed that the microbiome elements of the blepharitis and control groups are quite different from each other (Fig. 5).

**Fig. 6:**
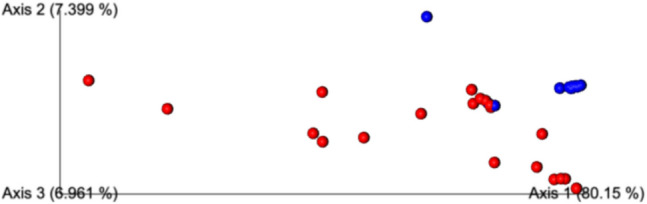
Beta diversity analysis according to Weighted Unifrac Index

In beta diversity analysis according to the Weighted Unifrac index; It is also seen that the similarity between the blepharitis and the control group is quite low, and the densities of different microorganisms are significantly different (80.15% of the PCo1 value) (Fig. 6).

**Fig. 7:**
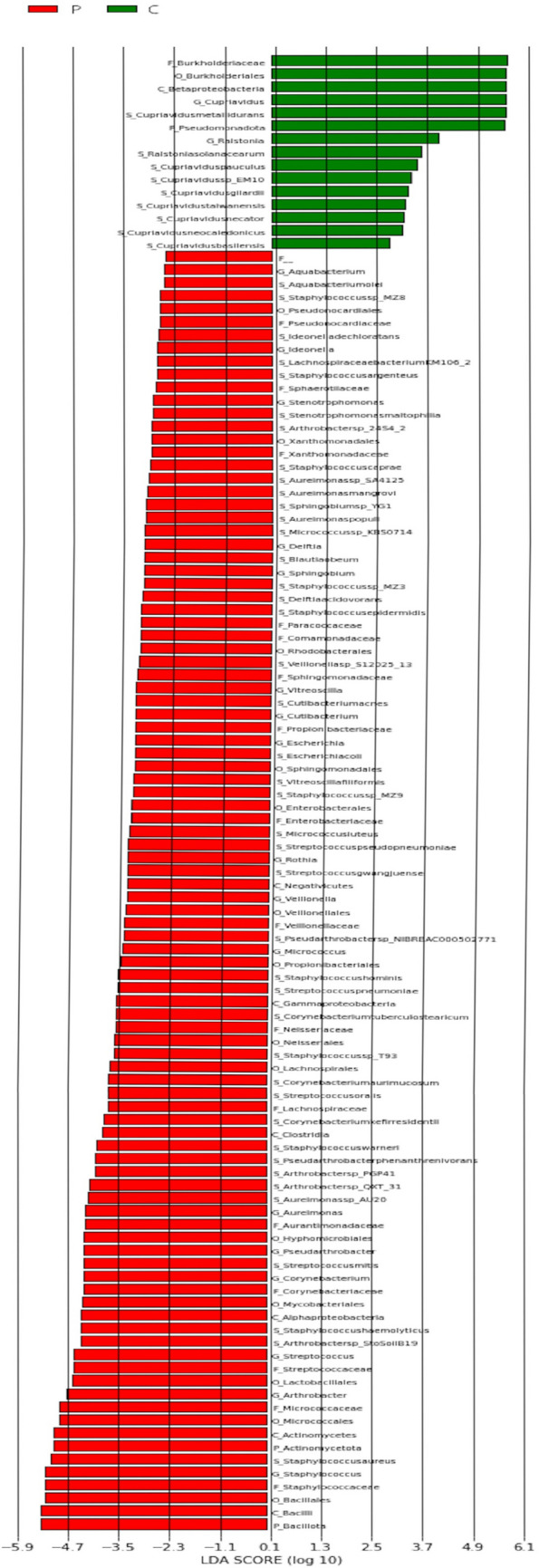
Genus-level LEfSe analysis of samples

LEfSe is an algorithm for biomarker discovery that identifies genomic features (genes, pathways or taxa) that characterize differences between metagroups. LEfSe first robustly identifies features that are statistically different between metagroups. It then performs additional tests to assess whether these differences are consistent with expected biological behavior. LEfSe uses LDA to estimate the effect size of each differentially abundant trait. Figure 7 shows the Genus-level LEfSe analysis of the samples. Table 3 reveals the distribution of bacteria at different levels.

**Table 3:** Classification of bacteria in two groups

	Blepharitis group	Control group
Phylum		*Pseudomonadota*
Class		*Betaproteobacteria*
Order	*Pseudonocardilaceae*	*Burkholderiales*
Family		*Burkholderiaceae*
Genus	*Aquabacterium*	*Cupriavidus, Raistonia*
Species	*Staphylococcus, Lacnospiracebacterium, Stenothermophilus, Aurelmonass, Sphingoblump, Micrococus,Blatiabeum, Delfiacdiovorans, S.epidermidis, Vellonella, Cutibacterium, Bacillus*	*Cupriavidusmetallduranse Ralstoniasollamacerum*

## Discussion

In this prospective study, conjunctival swab samples were obtained from pediatric patients with and without blepharoconjunctivitis and ocular microbiota were compared. Since antibiotic use has been shown to alter ocular microbiota, cessation of antibiotic use for at least six weeks was the inclusion criterion in both groups [8, 9].

The first description of ocular surface microbiota in the literature dates back to the 1930 s. At that time, *coagulase-negative staphylococci, Propionibacterium spp., Corynebacterium spp., Staphylococus aureus* and *Streptococcus* were thought to constitute the majority of the ocular surface microbiome [10]. With the development of sequence technologies, *P. aeruginosa, Haemophilus influenzae, Megasphaera elsdenii, Bacteroides ureolyticus, Bacteroides pneumosintes, Betaproteobacteria* and *Stomatococcus* species have been identified among some variations identified as common pathogens of the ocular microbiome [11].

According to studies in the literature, the majority of conjunctiva, skin, ocular surface and eyelid flora elements consist of *Pseudomonadota* family and *Cupriavidus* and *Ralstonia* species [12]. Although the ocular surface microbiome elements of healthy people found in our study are compatible with the literature, it is important that some new species were found in addition to the healthy eye flora elements in the literature. In a study examining ocular surface microbiota in children, it was shown that *Proteobacteria* dominated the flora [13]. In our study, it was observed that *betaproteobacteria* were significantly higher in the healthy control group at class level.

It has been observed that *Pseudonocardilaceae*, *Staphylococcus* and *Cutibacterium* species, which are dominant in the patient group with blepharitis, are also dominant species in diseases such as Trachoma and Sjögren's disease. Studies are needed to elucidate the role of these bacteria in the common mechanisms in the pathogenesis of both blepharitis and these diseases [14].

It has been reported that *Sphingoblump* and *Micrococus* species, which we found with high rates in the patient group, are also found predominantly in the ocular flora of patients with diabetes [15].

In another study in the literature in which ocular surface and meibomian gland microbial communities were examined in adult patients with Demodex blepharitis, it was found that the abundance of *Sphingoblump* species was higher, similar to our study. As a common result of this study and our study; it suggests that *Sphingoblump* species can be used as a bacterial biomarker in patients with blepharitis [16].

To our knowledge, there is no other study evaluated ocular surface microbiota in children with blepharoconjunctivitis with or without corneal involvement. On the other hand, Filippelli et al. demonstrated that probiotic supplementation reduces the time taken for complete resolution of the chalazion without inducing noteworthy complications and prevents its recurrence in the treated children [17]. Regarding chalaziosis, it is suggested that there is an association between probiotics, miRNAs and changes in the composition of the oil secreted from the Meibomian glands, which makes it less fluid [18]. Theoretically, the gut microbiota could interact by two different pathways; either via crosstalk between gut and ocular microbiota, or by the distant action on inflammation makers [19]. A higher rate of H. pylori has been observed in the stomach of patients with blepharitis compared to controls, and antibiotic eradication improved signs of blepharitis [20]. These studies revealed that gut microbiota, diet might alter ocular surface disease. In this current study, lifestyle of children could not be questioned. In further studies, this topic should be taken into account.

## Conclusions

With this prospective study, ocular microbiota in children with blepharoconjunctivitis was studied for the first time. Both alpha and beta diversity analyses were significantly different in this group compared to the healthy control group. Some bacteria, which have been detected on the ocular surface in adult blepharitis and some diseases affecting the ocular surface, were also seen intensively in the pediatric patient group. In addition, *Lacnospiracebacterium, Stenothermophilus, Aurelmonass, Micrococus, Blatiabeum, Delfiacdiovorans* and *Vellonella* densities were found to be higher, which may lead to future studies focused on diagnosis and treatment.

## Data Availability

The data that support the findings of this study are available from the corresponding author, Burçin Çakır, upon reasonable request.
